# Does the Use of Different Remineralisation Agents in a 14-day Demineralisation/Remineralisation Cycle Affect the Bond Strength to Artificial Carious Enamel Surfaces?

**DOI:** 10.3290/j.ohpd.c_1977

**Published:** 2025-06-03

**Authors:** Özgül Carti Dörterler, Fatma Yilmaz, Saniye Eren Halici, Aysegul Demirbas, Elif Yigit

**Affiliations:** a Özgül Carti Dentist, Department of Paediatric Dentistry, Faculty of Dentistry, Mugla Sitki Kocman University, Turkey. Idea, hypothesis, experimental design, performed the experiments in partial fulfillment of requirements for a degree: prepared the samples and artificial caries lesions, performed pH-cycling, restorative procedures, and SBS test; conducted SEM and EDX evaluations; evaluated fracture types. Wrote and proofread the manuscript, consulted on and performed statistical evaluation, contributed substantially to discussion.; b Fatma Yilmaz Dentist, Department of Restorative Dentistry, Faculty of Dentistry, Mugla Sitki Kocman University, Turkey. Idea, hypothesis, experimental design, performed the experiments in partial fulfillment of requirements for a degree: performed pH-cycling, restorative procedures, and SBS test; conducted SEM and EDX evaluations; evaluated fracture types. Wrote and proofread the manuscript, consulted on and performed statistical evaluation, contributed substantially to discussion.; c Saniye Eren Halici Dentist, Department of Prosthetic Dentistry, Faculty of Dentistry, Mugla Sitki Kocman University, Turkey. Idea, hypothesis, experimental design, performed the experiments in partial fulfillment of requirements for a degree: prepared the samples and artificial caries lesions; performed pH-cycling, restorative procedures, and SBS test; evaluated fracture types. Wrote and proofread the manuscript, consulted on and performed statistical evaluation, contributed substantially to discussion.; d Aysegül Demirbas Professor, Department of Restorative Dentistry, Faculty of Dentistry, Ege University, Turkey. Idea, hypothesis, experimental design, proofread the manuscript, contributed substantially to discussion.; e Elif Yigit PhD Student, Department of Chemistry, Faculty of Science, Mugla Sitki Kocman University, Mugla, Turkey. Experimental design, performed the experiments in partial fulfillment of requirements for a degree, prepared artificial caries lesions.

**Keywords:** adhesion, bond strength, remineralising agents, SEM-EDX analysis.

## Abstract

**Purpose:**

To examine the effect of applying different forms of remineralising agents during a pH-cyclinge on the bond strength of a universal adhesive applied in the etch-and-rinse mode and the types of fractures that occur after shear bond-strength testing.

**Materials and Methods:**

84 human molars were divided into seven equal groups. Groups 1 (intact enamel) and 2 (artificially demineralised enamel) served as the positive and negative controls, respectively. In the experimental groups (3-7), the enamel was treated using remineralising agents during a 14-day pH-cycling protocol. Group 3: fluoride gel; group 4: fluoride varnish; group 5: Tooth Mousse; group 6: MI Paste Plus; group 7: MI Varnish. Afterwards, the molars’ crowns were sectioned off, and a universal adhesive (G2-Bond Universal) was applied to the buccal surfaces of these samples using etch-and-rinse mode. Nanohybrid resin composite restorations (G-aenial Posterior) were then placed, and shear bond-strength testing was performed. The effects of remineralisation agents on artificial carious lesions were evaluated using scanning electron microscopy/energy-dispersive analysis.

**Results:**

The fluoride varnish and MI varnish groups show statistically significantly lower shear bond strengths compared to the positive control group (p<0.001). The bond strength to all remineralising agents was higher than that of the negative control group. All tested agents promoted remineralisation in demineralised areas of the enamel surface.

**Conclusions:**

Remineralisation agents in forms other than varnish do not negatively affect the shear-bond strength to enamel surfaces.

Epidemiological evidence has shown that dental caries is a highly prevalent chronic disease, affecting millions of people worldwide.^
16
^ The appearance of caries varies from non-cavitated lesions (such as white spots on enamel surfaces) to cavitated carious lesions (such as deep dentin cavities).^
20
^ Once caries begins, it is difficult to stop and formed cavitated lesions are irreversible.^
41
^ In the treatment of early-stage caries, remineralisation agents are used, while cavitated carious lesions require restorative procedures for treatment.

Fluoride is the most commonly used remineralising agent.^
30
^ Fluoride has a high affinity for calcified tissues.^
22
^ In the oral cavity, fluoride is released from dental materials and other sources when the pH drops, inducing the formation of fluorapatite or fluorohydroxyapatite and reducing acid solubility.^
12
^ When the pH rises, fluoride remineralises the enamel crystalline structure.^
48
^ It is important to assess this effect of fluoride on remineralisation, which influences the structure of hard dental tissues. This structure influences the tooth’s ability to perform its function, including withstanding occlusal forces, and can be evaluated by measuring its structural and mechanical properties.^
40
^


Casein phosphopeptide-amorphous calcium phosphate (CPP-ACP), a derivative of milk protein, is another remineralisation agent recommended for enamel remineralisation and caries prevention. CPP-ACP is a bioactive substance, where CPP – derived from the casein protein in milk – can stabilise calcium and phosphate in solution in nanocluster ion forms, thus helping increase the levels of calcium and phosphate in dental plaque,^
13
^ while ACP, through its localisation on the tooth surface, ensures saturation of the enamel structure with calcium and phosphate.^
14
^ Calcium and phosphate penetrate the enamel prisms and restore the apatite crystals. As a biologically active substance, ACP releases calcium and phosphate, preparing supersaturation, reducing demineralisation, and enhancing remineralisation. Calcium and phosphate ions easily diffuse through the porous carious lesion (both artificial and natural), precipitating onto the partially demineralised enamel prisms and reforming the apatite crystals.^
35
^ Since CPP-ACP plays an important role in reducing caries and is safe if swallowed by children under the age of two, it is widely used in paediatric dentistry, especially in high-risk children with poor oral hygiene.^
6
^


MI Paste Plus (GC; Tokyo, Japan) is a new product recommended for the treatment of early caries, containing CPP-ACP and 900 ppm fluoride. The simultaneous application of CPP-ACP and fluoride exhibits a synergistic effect. This product has shown promising results for caries control.^
26,36
^


MI Varnish (GC) provides a high dose of fluoride with an additional reinforcing effect from calcium and phosphate ions.^
7
^ While varnish application is recommended every three months for individuals with high caries activity, gel application is suggested for daily use.^
37
^


Changes in the mineral and protein structures of the superficial layer of enamel can affect the bond strength of restorative materials. Additionally, the composition of the remineralising agent and composite resins may lead to differences in bond strength.^
1
^ Strong, durable adhesion of restorative materials (such as composite resin) to tooth tissues is paramount for the success of the restoration.^
33
^ Adhesives are used to bond composite resins to tooth tissues. Their effectiveness and quality are crucial for establishing a stable connection between the composite and the hard dental tissues. In-vitro studies often evaluate the bond strength of composites to hard dental tissue using shear-bond strength (SBS) tests.^
27
^ Therefore, the SBS is an important measure of the effectiveness and quality of adhesives. Inadequate SBS can lead to early restoration failure under minimal chewing forces.^
22
^


Considering the common use of remineralisation agents and the potential need for restorations on teeth to which a remineralising agent has been applied, as well as the role of adhesive SBS in the success of composite restorations, it is essential to evaluate the effect of remineralisation agents on the SBS of dental adhesives.

The primary aim of this in-vitro study was to examine the effect of applying different forms of remineralising agents during pH-cycling on the bond strength of a universal adhesive applied in the etch-and-rinse mode and the types of fractures that occur after SBS testing.

The null hypothesis of the study is that there is no difference in SBS between the adhesion of the resin composite using a universal adhesive to intact and demineralised enamel after the application of the tested remineralising agents.

## Materials and Methods 

### Power Analysis

In this in-vitro study, human molars extracted from the maxilla or mandible for orthodontic and periodontal reasons were used. Seven experimental groups were formed, and a power analysis was conducted using G Power software to determine the sample size required for the statistical comparison of bond strengths between the groups. With a 95% confidence level (1-α), an effect size of f = 0.5, and 80% test power (1-β), the minimum number of samples required per group was determined as 9. Therefore, the sample size was set at n = 10 for each group. Additionally, two tooth samples were included in each group for SEM/EDX (Scanning Electron Microscope/ Energy Dispersive X-Ray) analysis to analyse and characterise the effectiveness of remineralisation agents.

### Preparation of Samples

Eighty-four caries-free molars collected for orthodontic and periodontal purposes were dried with an air spray and visually examined to exclude teeth with visible structural defects, cavitated lesions, visible white spot lesions, and/or colour changes. Soft tissue residues on the teeth were gently curetted and cleaned with fluoride-free pumice, polishing brushes, and fluoride-free water. The samples were stored in a 0.1% thymol solution (ADR Group; Istanbul, Turkey) until use. The teeth were used within six months of extraction in accordance with ISO standards.^
28
^ For the shear bond-strength test, the buccal surfaces of 10 molars were slightly polished using 320-grit and 600-grit silicon carbide abrasive papers with water lubrication to create flat enamel surfaces. The two molars to be used in the EDX analysis were not treated in order to avoid disrupting the surface integrity of the enamel tissue.

### Study Groups

Eighty-four teeth were randomly divided into seven groups of n = 12 teeth (2 for the remineralisation efficacy assessment using SEM/EDX and 10 for bond-strength testing), using a random numbers table. The groups were designed as follows:

Group 1: intact enamel (positive control) + artificial salivaGroup 2: demineralised enamel (negative control) + artificial salivaGroup 3: demineralised enamel + fluoride gelGroup 4: demineralised enamel + fluoride varnishGroup 5: demineralised enamel + Tooth MousseGroup 6: demineralised enamel + MI Paste PlusGroup 7: demineralised enamel + MI Varnish

All materials used in the study are presented in Table 1.

**Table 1 table1:** Commercially available materials used in this study

Material	Composition	LOT number	Product name; manufacturer
Fluoride gel	1.23% acidic phosphate fluoride	20A249	Polimo 1.23% APF gel; Imicryl, Konya, Turkey
Fluoride varnish	5% sodium fluoride	20065	Polimo Fluoride varnish; Imicryl
CPP-ACP	Casein phosphopeptide-amorphous calcium phosphate%10,0	7370418	Tooth Mousse; GC International; Tokyo, Japan
CPP-ACP-Fluoride	Casein phosphopeptide-amorphous calcium phosphate, sodium fluoride 0.2% w/w (900 ppm), purified water, glycerol, d-sorbitol, CMC-Na, propylene glycol, silicon dioxide, titanium dioxide, xylitol, phosphoric acid, flavouring agent, ethyl-, propyl-, and butyl-p-hydroxybenzoate	1107261	MI paste plus; GC
CPP-ACP–Fluoride varnish	30–50% polyvinyl acetate, 10–30% hydrogenated pine rosin, 20–30% ethanol, 5% sodium fluoride, 5% CPP-ACP, 1–5% silicon dioxide	1412172	MI Varnish; GC
Universal bonding agent	Primer: 10-MDTP, 10-MDP, 4-MET, acetone, water, initiators, fillers, water Bond: bis-GMA, dimethacrylate monomer, filler, photoinitiator	Primer: 2210071 Bond: 2210111	G2-BOND Universal; GC
Composite resin material	A hybrid composite comprising a combination of pre-polymerised resin filler (silica, strontium, and lanthanides) and inorganic filler (fluoroaluminosilicate > 100 nm; fumed silica < 100 nm) with a matrix of UDMA and dimethacrylate co-monomers	1211062	G-aenial composite; GC
Artificial saliva	NaCl, KCl, CaCl_2_, Na CMC (C_8_H_15_NaO_8_) MgCl_2_, aspartame C_14_H_18_N_2_O_5_, K_2_HPO_4_	SAE0149	Sigma Aldrich: St. Louis, MO, USA
Lactic acid	DL-lactic acid, 2-hydroxypropanoic acid	-	ERBAPharmBP-DAB-FU Ph.Eur; Val de Reuil, France


### Artificial Caries Formation 

At the initial stage, except for the 12 teeth in the positive control group, the remaining samples were immersed in 0.1 M lactic acid adjusted to pH 4.4 for 72 h to induce artificial caries-like lesions.^
23,45
^ After the artificial caries lesions were formed, all teeth except for those in the positive and negative control groups were subjected to pH-cycling in a 37°C shaking water bath to mimic the oral environment.

### pH-Cycling (Demineralisation–Remineralisation)

pH-cycling involved immersing the samples in a demineralising solution with pH 4.4 (0.1 M lactic acid) for 6 h (30 ml per group), followed by treatment with the specified remineralising agent for 120 s and then keeping the samples in artificial saliva for 18 h.^
23,31,49
^ To remove potential residues between the demineralisation and remineralisation cycles, the teeth were washed with deionised water. The demineralising solution and artificial saliva solution were changed daily. pH-cycling was continued for 14 days. At the end of this time, two molars from each group were selected for SEM/EDX analysis.

### Evaluation of Surface Topography Using SEM 

The samples randomly selected from each group were gold-coated and then analysed using a scanning electron microscope (Quanta FEG 250, FEI; Hillsboro, OR, USA). SEM images were examined to evaluate the surface damage after artificial caries lesion formation and the micromorphology of the enamel surface following the application of a remineralisation agent under simulated oral conditions.

### Surface Composition Analysis Using EDX

To determine the remineralisation rate on the surface of the treated samples, the concentrations of calcium (Ca), phosphorus (P), and fluoride (F) ions were analysed and compared with those of untreated samples. For this purpose, an EDX analysis was applied to full area with an S-UTW detector (EDAX; Mahwah, NJ, USA) using an EDX device (EVO MA 10, Zeiss; Oberkochen, Germany), in conjunction with the scanning electron microscope.

### Restorative Procedure

The roots of the remaining teeth in the groups were sectioned off from the crowns at the cementoenamel junction using a precision cutting device (Metkon; Bursa, Turkey) under water cooling. All buccal surfaces of molar crowns were dried with compressed air. In the groups where varnish was applied, polishing was performed using a polishing rubber attached to a contra-angle handpiece to remove the varnish from the surface. The polished teeth were then placed in an ultrasonic bath for 30 min to eliminate any remaining varnish on the enamel surface.^
32
^ The adhesive (G2-BOND Universal) was applied in etch-and-rinse mode to the buccal surface using a bonding jig according to the manufacturer’s instructions. In the first stage, the primer was applied to a single layer, allowed to sit for 10 s, and then dried with maximum air for 5 s. After that, the bond was applied, gently dried with air to achieve a uniform surface, and polymerised for 10 s. Teflon molds with a diameter of 4 mm and a height of 4 mm were then placed on the samples, and A2 shade composite resin (GC, Tokyo, Japan) was applied and polymerised using a Bluephase Style LED (Ivoclar Vivadent; Schaan, Liechtenstein) with an intensity of 1100 mW/cm^
2
^. The teeth were then stored in distilled water at 37°C for 24 h.

### SBS Testing

**Fig 1 Fig1:**
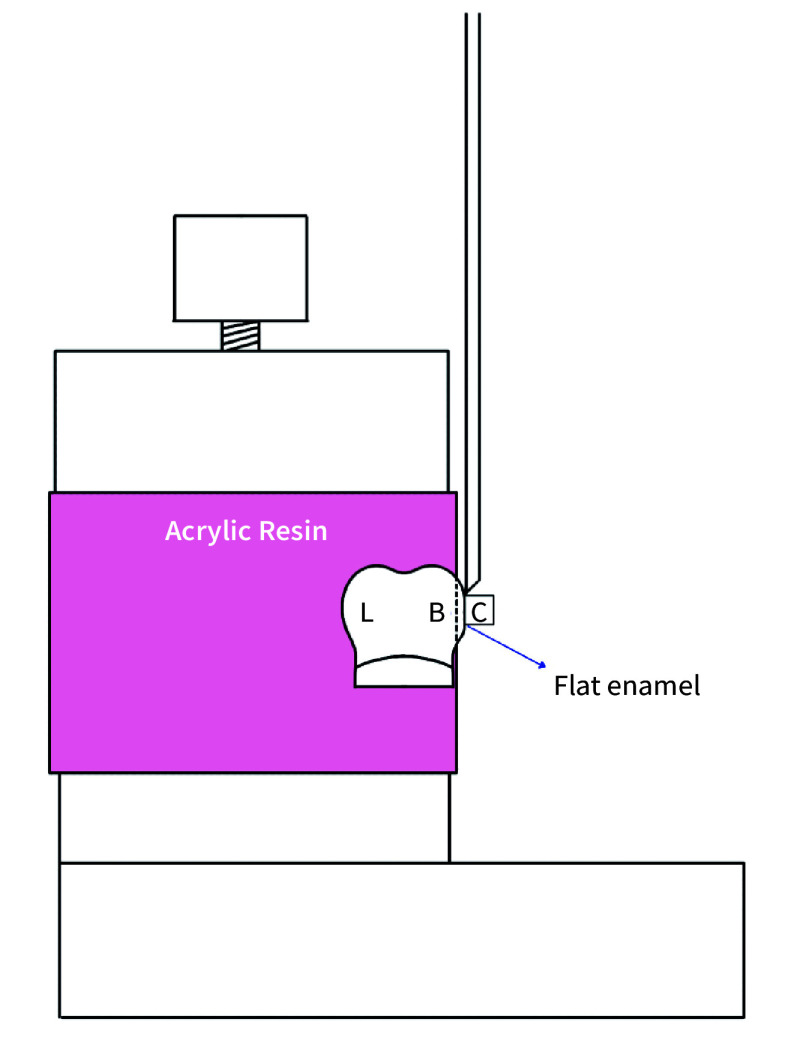
Schematic representation of the SBS test (B: buccal surface; L: lingual surface; C: composite resin).

After being stored in distilled water at 37°C for 24 h, the restored teeth were placed into cylinders with an inner diameter of 15 mm and a height of 20 mm. SBS was tested using a universal testing machine (Shimadzu AGS-J; Tokyo, Japan). The cylinders were positioned vertically, and the force was applied perpendicular to the composite–tooth interface in the incisal-cervical direction using a steel tip with a 30-degree angled termination. The load was applied at 1 kN with a crosshead speed of 1 mm/min. The force required to break the composite material was recorded in Newtons (N) and converted to megapascals (MPa) by dividing N by the bonding area (12.56 mm^
2
^) (MPa = N/mm^
2
^). A schematic representation of the SBS test is shown in Fig 1.

### Evaluation of Fracture Types

The fracture surfaces were examined under a stereomicroscope (SMZ-U ZOOM 1:10 Nikon; Tokyo, Japan) to determine the fracture mode and categorised into one of the following four types: 1. cohesive fracture in enamel; 2. cohesive fracture in composite; 3. adhesive fracture; 4. mixed fracture (partially cohesive and partially adhesive).

### SEM Analysis of Fracture Types

For a detailed evaluation of the fracture surfaces, one fracture type from each group was selected and examined using SEM (FEI). The crowns were cleaned with distilled water spray, dried, and left at room temperature for 24 h for dehydration. The surfaces were then coated with a thin layer of gold (BIO-RAD Polaron SEM Coating System; Hempstead, UK) and placed in a vacuum chamber with a voltage of 2.5 kV and a current of 20 mA. The teeth were then examined at 10 kV using the microscope.

### Statistical Analysis

Data were analysed using IBM SPSS V23. The normality of the data distribution was assessed using the Shapiro-Wilk test. For comparisons of non-normally distributed data among three or more groups, the Kruskal-Wallis H test was used, and multiple comparisons were examined with the Dunn test. The analysis results were presented as mean ± standard deviation (SD) and median (minimum–maximum). The significance level was set at p < 0.05.

## Results

### Shear Bond Strength Testing

The lowest SBS was observed in the negative control group. The fluoride varnish and MI Varnish groups showed statistically significantly lower SBS compared to the positive control group. When comparing the remineralisation agents, fluoride varnish exhibited statistically significantly lower bond strength to composite than did fluoride gel. There were no statistically significant differences in bond strength among the other remineralisation agents (Table 2).

**Table 2 table2:** Comparison of maximum stress values in MPa by group

Group	Bond strength	Test statistics	p*
Mean ± SD
Positive control	16.13 ± 2.6^c^	32.09	<0.001
Negative control	6.59 ± 2.86^d^
Fluoride gel	15.18 ± 2.4^bc^
Fluoride varnish	10.61 ± 2.28^a^
Tooth Mousse	12.35 ± 2.5^abc^
MI Paste	14.09 ± 1.3^abc^
MI Varnish	11.08 ± 2.6^ab^
*Kruskal-Wallis H-test; Different superscript letters represents statistically significant differences.

**Fig 2 fig2:**
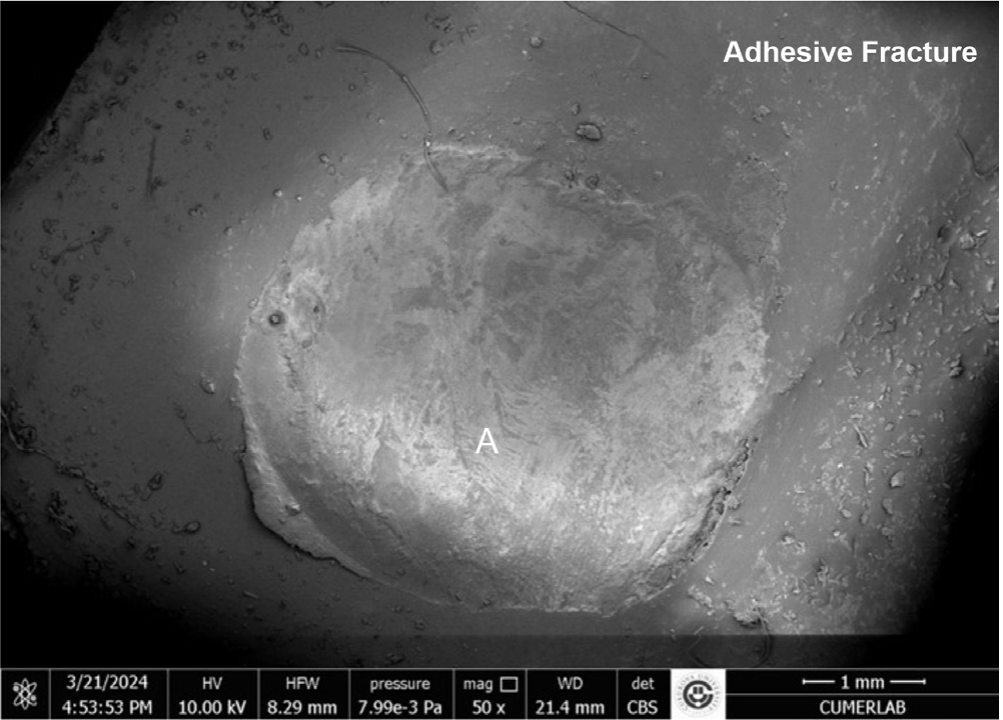
SEM image of fluoride varnish group, adhesive fracture (A: adhesive remnant).

**Fig 3 fig3:**
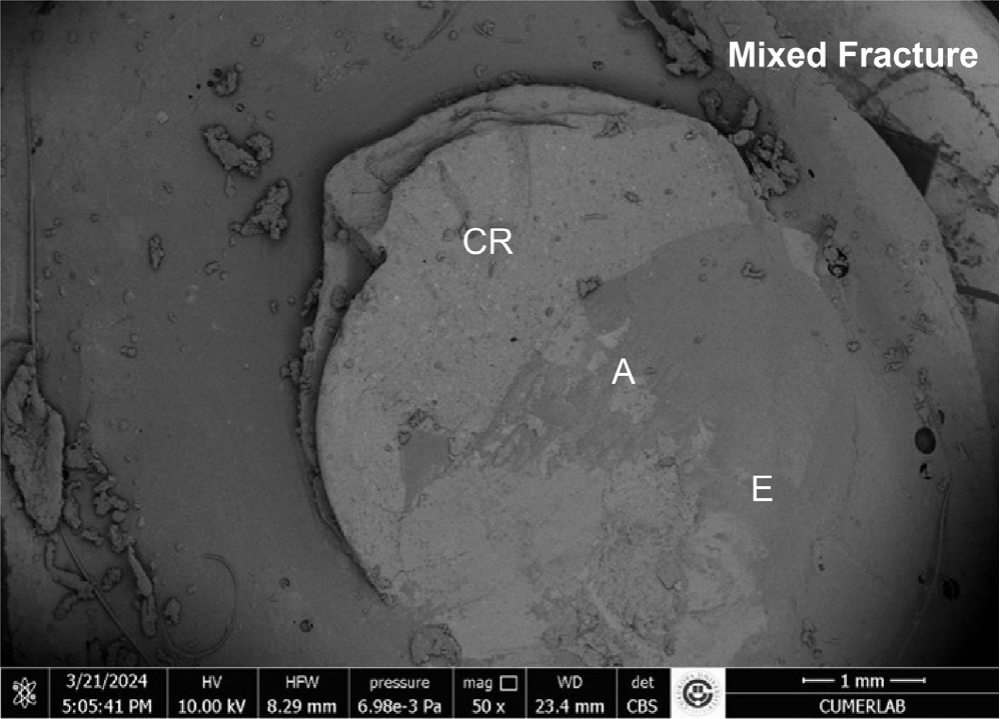
SEM image of fluoride gel grou, mixed fracture (A: adhesive remnant; E: enamel; CR: composite resin).

**Fig 4 fig4:**
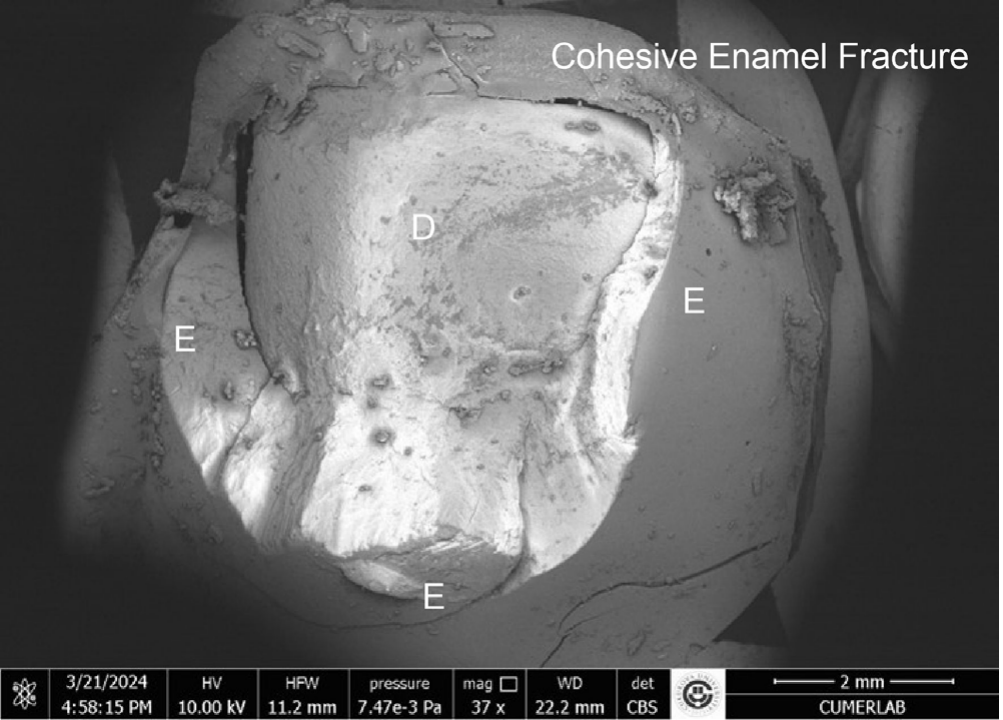
SEM image of the Tooth Mousse group, cohesive enamel fracture (E: enamel, D: dentin).

**Fig 5 fig5:**
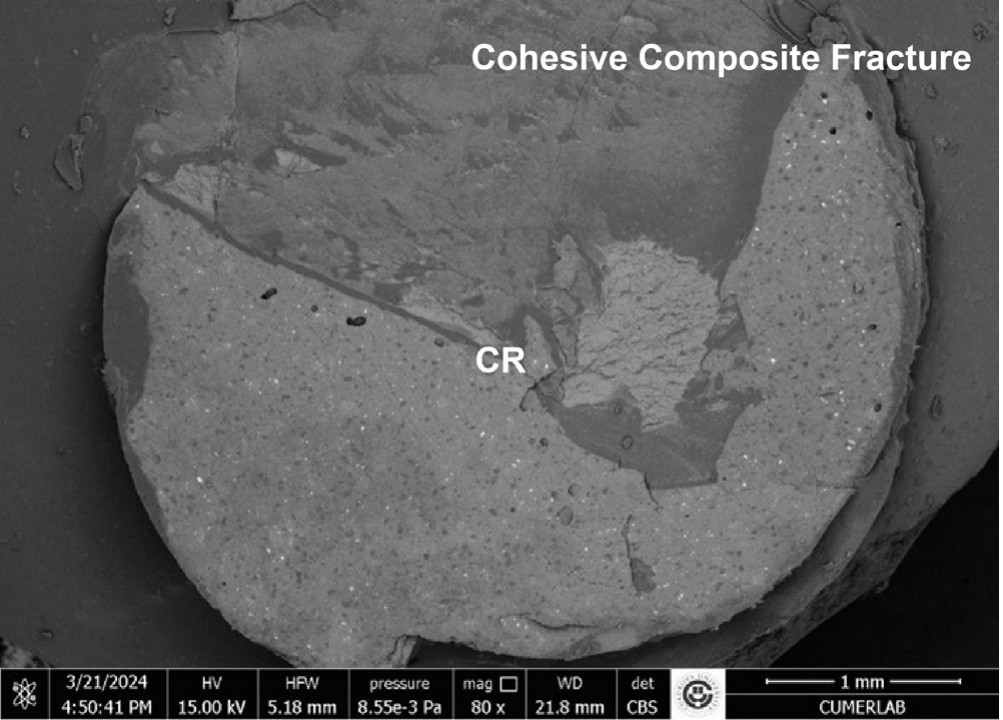
SEM image of positive control group, cohesive composite fracture (CR: composite resin).

The results of the stereomicroscopic evaluation of fracture mode are summarised in Table 3. The most commonly observed fracture type in the fluoride varnish and MI Varnish groups was adhesive (Fig 2), and that in the fluoride gel group was mixed (Fig 3). In the Tooth Mousse and MI Paste Plus groups, the rate of cohesive enamel fractures (Fig 4) was higher than that of the other fracture types. Cohesive composite fractures occurred most frequently in the positive control group (Fig 5).

**Table 3 table3:** Fracture distributions of groups

Group	Adhesive	Cohesive in enamel	Cohesive in composite	Mixed
Positive control	0%	0%	30%	70%
Negative control	10%	20%	0%	70%
Fluoride Gel	10%	0%	0%	90%
Fluoride Varnish	60%	0%	0%	40%
Tooth Mousse	20%	40%	0%	40%
MI Paste Plus	0%	50%	0%	50%
MI Varnish	60%	0%	0%	40%


### Surface Topography Analysis (SEM Analysis)

SEM showed that the different remineralisation agents applied to artificial carious lesions had correspondingly different morphological appearances.

**Fig 6 fig6:**
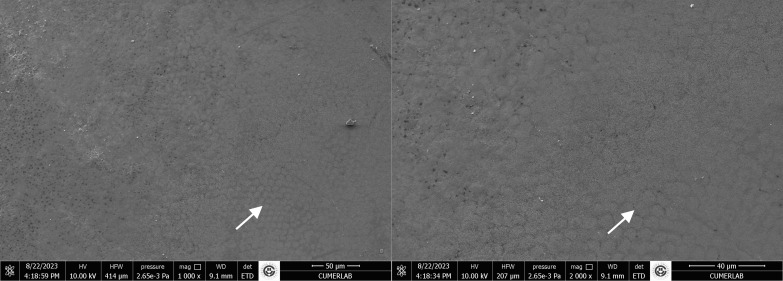
SEM images of positive control group at 1000X and 2000X magnifications. Arrows indicate the hexagonal crystallite structure of the enamel prisms.

In the positive control group, the enamel surface appeared smooth, with well-preserved enamel crystals. The characteristic orderly structure of an intact enamel crystal was visible, and the hexagonal crystallite structure of the enamel prisms was clearly observed (Fig 6).

**Fig 7 fig7:**
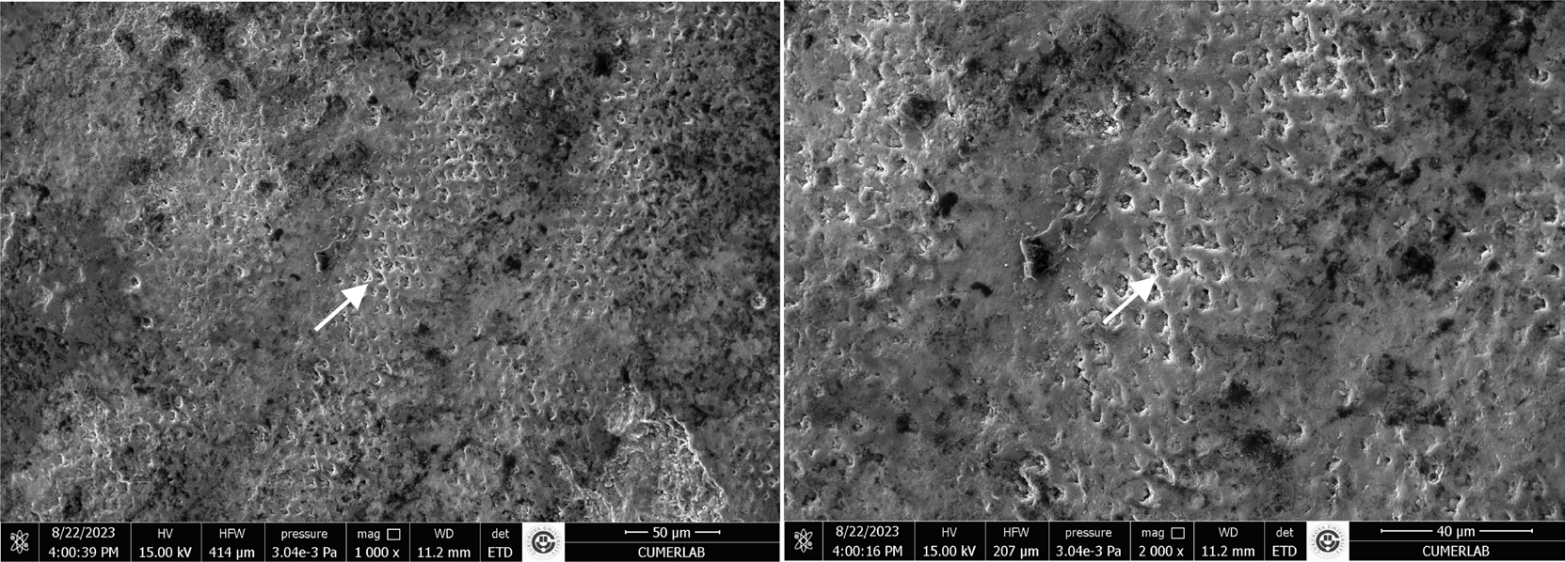
SEM images of negative control groupat 1000X and 2000X magnifications. Arrows indicate the porous structure of the enamel prisms with disrupted integrity.

Conversely, in the negative control group – bearing artificial caries lesions – showed a porous structure characterised by keyhole-shaped enamel prisms, which are typical features of decayed enamel. The integrity of the enamel prisms was severely compromised, with irregular surfaces and disruptions present in the interprismatic region (Fig 7).

**Fig 8 fig8:**
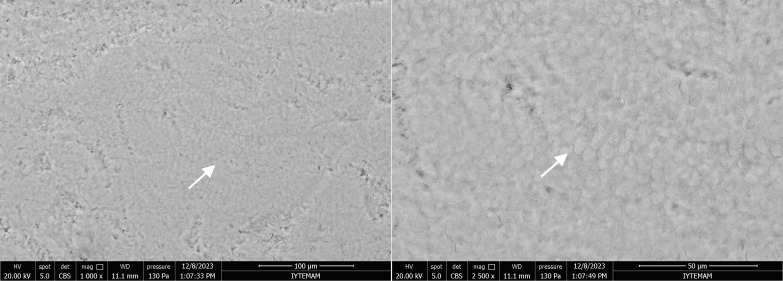
SEM images of the enamel sample from the fluoride gel group at 1000X and 2500X magnifications. Arrows indicate a decrease in surface porosity and an enhancement in the enamel crystal structure.

Following the 14-day demineralisation–remineralisation cycle, all experimental groups demonstrated a decrease in surface porosity and an improvement in enamel crystal structure. In the fluoride gel group, the enamel surface showed signs of reorganisation through remineralisation along the prismatic boundaries. Improvements in the enamel crystal structure were observed. It was also noted that the gaps in the interprismatic region had closed (Fig 8).

**Fig 9 fig9:**
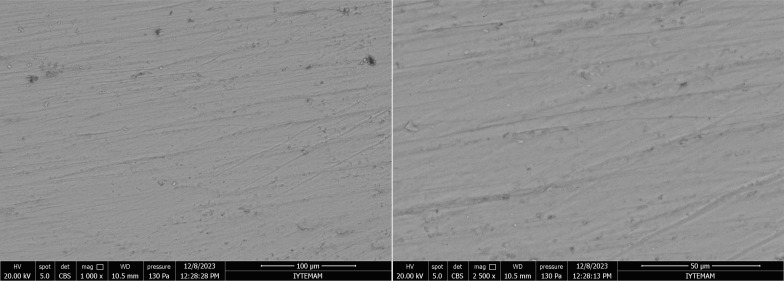
SEM images of the enamel sample from the fluoride varnish group at 1000X and 2500X magnifications. Note that the artificial caries lesions on the enamel surface have disappeared.

In the fluoride varnish group, the artificial caries lesions were completely eradicated, and the enamel surface exhibited even smoother characteristics than did the positive control group (Fig 9).

**Fig 10 fig10:**
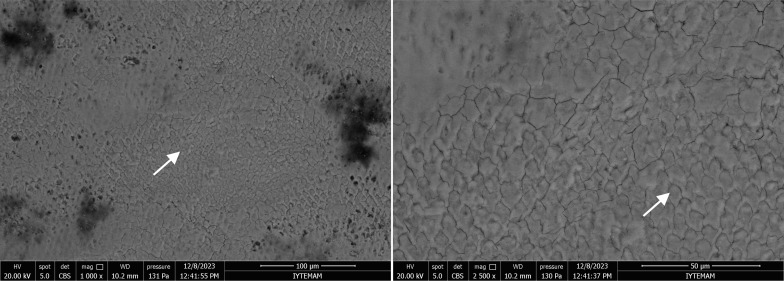
SEM images of the enamel sample from the Tooth Mousse group at 1000X and 2500X magnifications. Arrows indicate the areas of the porous structure densely filled with spherical calcium layers.

After the application of Tooth Mousse, the pores were observed to be densely and unevenly filled with irregular calcium layers and spherical formations. The porous defects were plugged, resulting in a reduction in cavities and micropores, and the re-establishment of surface integrity began. The cracks identified in these groups likely result from the rapid dehydration of the samples when exposed to the high vacuum conditions of the SEM (Fig 10).

**Fig 11 fig11:**
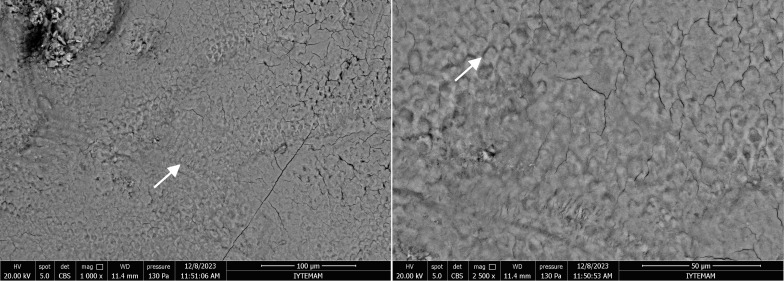
SEM images of the enamel sample from the MI Paste group at 1000X and 2500X magnifications. Note that the structure of enamel prisms has improved.

In the MI Paste group, the prismatic and interprismatic structures of the enamel were more pronounced, though areas of demineralisation remained present. The cracks identified in this group are also attributable to the rapid dehydration of the samples when exposed to the high vacuum conditions of the SEM, similar to those in the Tooth Mousse group (Fig 11).

**Fig 12 fig12:**
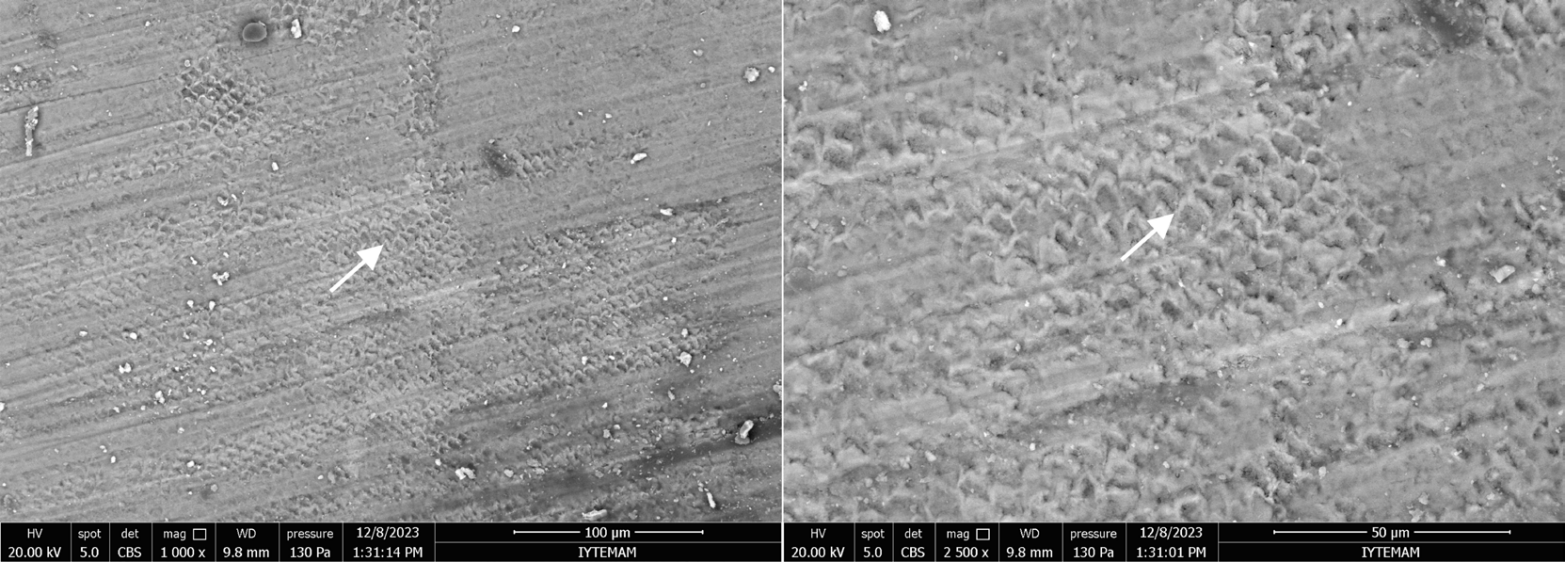
SEM images of the enamel sample from the MI Varnish group at 1000X and 2500X magnifications. Arrows indicate improvement in the porous structure caused by the artificial caries.

In the MI Varnish group, although the porous structure caused by demineralisation showed improvement, small pores were still present. Amorphous calcified deposits were scattered on the surface along the edges of the prisms after remineralisation (Fig 12).

### Surface Composition Analysis (EDX analysis) 

**Fig 13 fig13:**
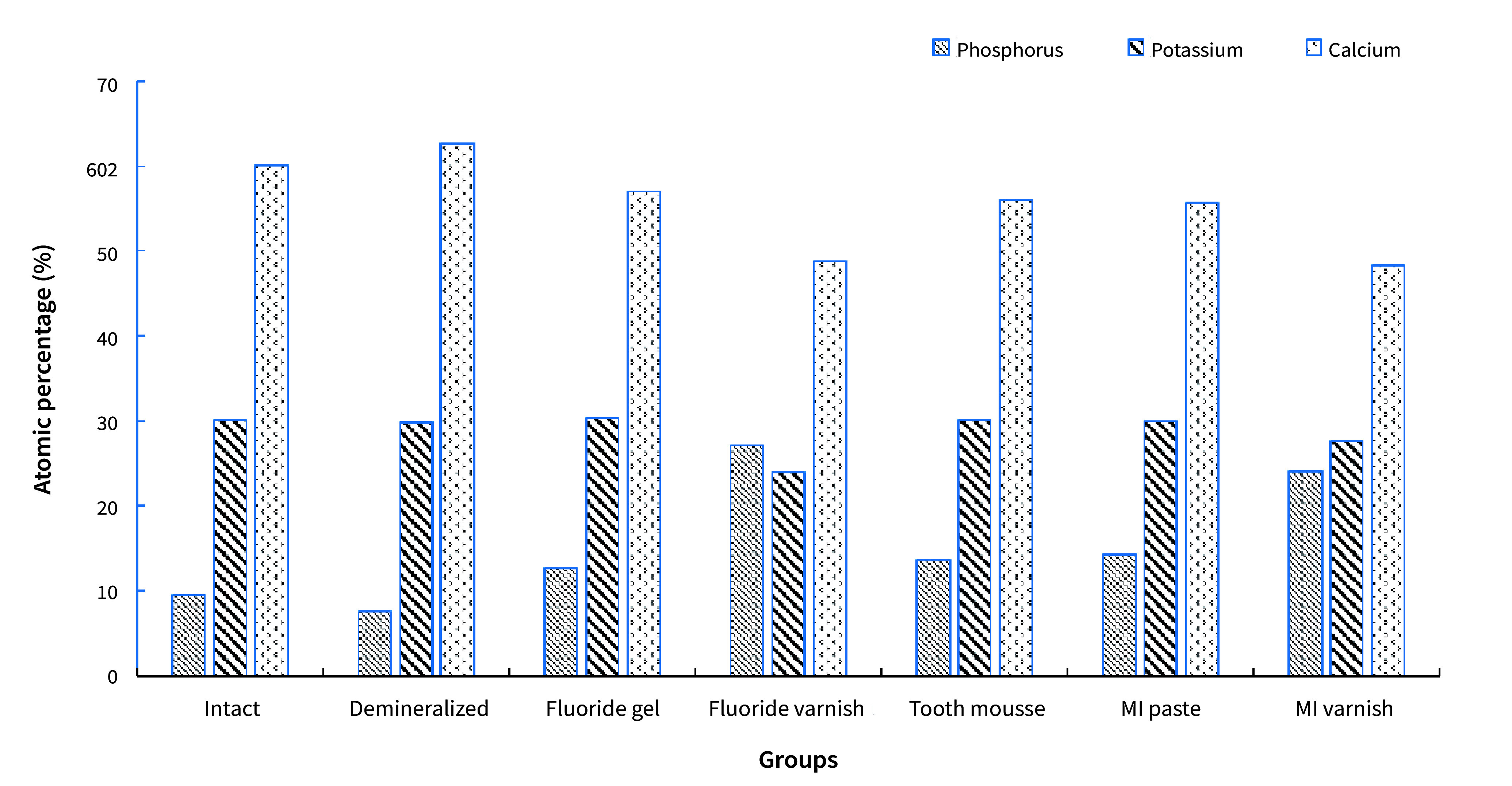
Atomic percentage distributions of ions for all groups.

Artificial caries formation resulted in a decrease in the concentrations of fluoride and phosphorus; the concentration of these minerals increased with the application of Tooth Mousse and MI Paste (Fig 13). The atomic percentages of calcium and phosphorus ions and the Ca:P ratio are presented in Table 4. In the fluoride varnish and MI Varnish groups, the lowest values were obtained for calcium and phosphorus, while the highest values were obtained for fluoride.

**Table 4 table4:** Atomic percentages of calcium and phosphorus ions and the Ca:P ratio according to the EDX analysis

	Positive control	Negative control	Fluoride gel	Fluoride varnish	Tooth Mousse	MI Paste Plus	MI Varnish
Ca	62.6±0.63	60.07±0.68	55.6±1.99	53.79±6.97	58.33±3.26	56.34±0.88	51.78±4.81
P	29.83±0.96	30.4±1.20	30.07±0.39	26.39±3.37	30.03±0.13	29.97±0.05	28.53±1.34
Ca:P	2.09	1.97	1.84	2.03	1.94	1.87	1.81


## Discussion

The aim of this in-vitro study was to evaluate the changes on the enamel surface caused by different remineralisation agents after creating artificial caries-like lesions on dental samples and simulating the oral environment, as well as to examine the shear bond strength of the universal adhesive applied to treated enamel surfaces.

The pH-cycling model is commonly used to investigate the effects of anti-caries agents on the dynamics of enamel demineralisation and remineralisation.^
19
^ In various studies, pH-cycling has been conducted for different durations, ranging from 7 to 14 days.^
18,34,46
^ For example, Ruiz et al^
32
^ suggested that for situations in which adhesive application is necessary, it is appropriate to apply the remineralising agent at least seven days before the bonding procedure. Therefore, in this study, we conducted pH-cycling period for 14 days, similar to previous studies.

In this study, a SBS test was conducted to determine the effect of the tested remineralisation agents on the bond strength of a universal adhesive to demineralised enamel. Numerous techniques and materials have been investigated in the literature to test the impact of remineralisation agents on the structural and mechanical properties of enamel. In this study, the agents evaluated were 1.23% APF fluoride gel, 5% NaF fluoride varnish, CPP-ACP paste, CPP-ACP + fluoride paste, and CPP-ACP + fluoride varnish. The results presented in the literature regarding the effect of different remineralising agents on SBS are inconsistent.^
24,42,44,51
^


Enamel tissue is composed of HAp, a hard solid crystalline structure with a high-energy surface and strong intermolecular forces in addition to water and organic material. HAp crystals constitute the majority of the inorganic content of enamel and dissolve under the influence of acids or acidic monomers during adhesion, leading to their removal from the oral environment.^
4
^ The resulting microporosities play a key role in the formation of micromechanical bonding. Similarly, when the pH of the environment shifts towards acidity, the caries process begins, resulting in enamel demineralisation, which involves the dissolution of HAp crystals. However, the caries process is a complex phenomenon that involves not only mineral loss but also the degradation of the organic matrix.^
29
^ The SEM images obtained in this study show that the prismatic structure of the enamel is disrupted, and the surface becomes more irregular and porous. This condition negatively affects adhesion to demineralised enamel, as the resin tags necessary for proper adhesion do not form in sufficient quantity or quality.^
10
^ Additionally, in this study, the SBS of demineralised enamel were found to be lower than those of sound enamel. Similarly, other studies have reported that enamel surface demineralisation reduces SBS.^
39
^


When fluoride is applied to an intact or demineralised area at neutral pH, a loosely bound CaF_2_ layer forms on the surface, which subsequently dissolves, releasing fluoride to react with calcium and phosphate ions. The final product of these reactions is fluorapatite, which prevents demineralisation and enhances the remineralisation of crystals.^
21
^ The formed fluorapatite is more resistant to acidic dissolution and micromechanical adhesion than is non-fluoridated Hap.^
32
^


In this study, the SBS of the groups treated with fluoride varnish and MI varnish were found to be statistically significantly lower compared to the sound group but higher than the negative control group (p < 0.001). Therefore, the null hypothesis of the study was rejected. This can be explained as follows: In the demineralisation–remineralisation cycle, the fluoride ion saturation of the tooth and its environment is crucial. A demineralised enamel surface is undersaturated with fluoride. When fluoride is applied, it precipitates onto the tooth surface in a higher amount compared to intact enamel, forming more acid-resistant fluorohydroxyapatite. Additionally, the varnish form of fluoride creates a continuous barrier on the enamel surface, which negatively affects adhesion. Supporting these results, the EDX analysis showed that the atomic percentage of fluoride ions was higher in the fluoride varnish and MI varnish groups compared to the other groups. Similarly, fluoride application in aqueous environments negatively impacts enamel bond strength.^
38
^ In another study, it was reported that fluoride varnish applied to demineralised enamel did not reduce the SBS to enamel.^
32
^ While bovine incisors were used in that study,^
32
^ human molars were preferred in our study. Although bovine teeth are often used as substitutes for human teeth in in-vitro studies, they have some structural differences. In bovine teeth, the enamel prisms are longer and harder.^
2
^ Due to this structural difference, Ortiz et al^
32
^ concluded that there was no decrease in bond strength to the demineralised enamel surface treated with varnish.

In this study, the SBS of the group treated with 1.23% APF fluoride gel were similar to those of the intact-enamel group and higher than those of the 5% NaF fluoride varnish group. The reason for this may be that, due to the low pH of the APF fluoride gel (~3.2), fluoride ions not only accumulate on the surface but also infiltrate into the subsurface layers. Consequently, the fluoride deposited in the enamel shows a more homogeneous distribution, leading to remineralisation of the enamel’s prismatic structure, similar to intact enamel. This is evident in the SEM images obtained in this study. As a result, less resistant fluorohydroxyapatite forms on the surface, leading to higher bond strength compared to the varnish group.

In this study, lower SBS were also obtained in the MI Varnish group compared to the intact-enamel group. This can be explained by the possibility that the MI Varnish led to greater retention of calcium and phosphate ions on the tooth surface, which might have disrupted the bonding mechanism. Similarly, Bayraktar et al^
8
^ reported lower bond strength in the MI Varnish group compared to the intact group.

Additionally, different methods have been used to determine the effectiveness of remineralising agents on artificial carious lesions. In the literature, the characterisation of the morphological features of artificial carious lesions at the initial stage and of remineralised enamel surfaces is often achieved using SEM.^
5
^ The present SEM observations showed noticeable improvements in the enamel crystal structure and a reduction in porosity in all groups treated with remineralising agents. EDX microanalysis is an analytical technique integrated with electron microscopy, enabling elemental analysis by detecting characteristic x-rays emitted by the specimen. This method provides detailed information about the elemental composition of the material under investigation.^
15
^ This technique can be applied to analyse tooth mineral content. It measures the atomic percentage of calcium (Ca) and phosphorus (P) on sound, demineralised, and remineralised enamel surfaces.^
45
^ In this study, an increase in the atomic percentages of Ca, P, and F ions was detected in the groups treated with remineralisation agents.

According to the literature, adhesion can be analysed in detail using SEM to determine its clinical performance.^
17
^ If the adhesion of the adhesive resin to dental tissue is strong enough, cohesive fractures may occur due to the decrease in the material’s cohesive strength.^
3
^ In this study, adhesive fractures were more prevalent in the fluoride varnish and MI Varnish groups compared to other fracture types. As similarly stated in the literature,^
8,25,43
^ the higher incidence of adhesive fractures is attributed to fluoride forming an acid-resistant layer on the enamel surface and the varnish residues creating a barrier that prevents acid penetration into the enamel prisms. In this study, an increase in the rate of cohesive enamel fractures was observed in the groups treated with Tooth Mousse and MI Paste Plus, compared to the other groups. The occurrence of cohesive fractures in the enamel surface indicates that the bond strength of the composite resin to the enamel surface was quite high. This result, in line with previous studies, demonstrates that the application of CPP-ACP before adhesive application is an effective method for enhancing the bond strength of resins to enamel.^
9,11
^


The most important limitation of this study is that in-vitro remineralisation conditions may differ considerably from those of the dynamic and complex biological systems of the oral cavity in-vivo. Therefore, different interpretations might be required when evaluating the applicability of these findings to clinical conditions.

## Conclusion

All tested agents promoted remineralisation in demineralised areas of the enamel surface. Compared to the demineralised negative control group, all remineralising agents enhanced the shear bond strength of the resin composite to the enamel surface. Fluoride varnish and MI varnish showed statistically signficantly lower bond strength than the positive control, while other remineralisation agents exhibited bond strength similar to that of the positive control.

## References
